# Liquid-Phase Partial Hydrogenation of Phenylacetylene at Ambient Conditions Catalyzed by Pd-Fe-O Nanoparticles Supported on Silica

**DOI:** 10.3390/nano13152247

**Published:** 2023-08-03

**Authors:** Anastasiya A. Shesterkina, Olga A. Kirichenko, Olga P. Tkachenko, Alexander L. Kustov, Leonid M. Kustov

**Affiliations:** 1 Chemistry Department, Lomonosov Moscow State University, Leninskie Gory 1/3, 119234 Moscow, Russia; anastasiia.strelkova@mail.ru (A.A.S.); kyst@list.ru (A.L.K.); 2 Laboratory of Nanochemistry and Ecology, Institute of Ecotechnologies, National University of Science and Technology MISIS, Leninsky Prospect 4, 119049 Moscow, Russia; 3 Laboratory of Development and Research of Polyfunctional Catalysts, Zelinsky Institute of Organic Chemistry, Russian Academy of Sciences, Leninsky Prospekt 47, 119991 Moscow, Russia; okiriche@ioc.ac.ru (O.A.K.); ot113@mail.ru (O.P.T.)

**Keywords:** alkyne selective hydrogenation, Pd-Fe catalysts, DRIFT spectroscopy, CO adsorption

## Abstract

Catalysts with no hazardous or toxic components are required for the selective hydrogenation of acetylenic bonds in the synthesis of pharmaceuticals, vitamins, nutraceuticals, and fragrances. The present work demonstrates that a high selectivity to alkene can be reached over a Pd-Fe-O/SiO_2_ system prepared by the co-impregnation of a silica support with a solution of the metal precursors (NH_4_)_3_[Fe(C_2_O_4_)_3_] and [Pd(NH_3_)_4_]Cl_2_ followed by thermal treatment in hydrogen or in air at 400 °C. A DRIFT spectroscopic study of CO adsorption revealed large shifts in the position of the Pd^n+^-CO bands for this system, indicating the strong effect of Fe^n+^ on the Pd electronic state, resulting in a decreased rate of double C=C bond hydrogenation and an increased selectivity of alkyne hydrogenation to alkene. The prepared catalysts consisted of mono- and bimetallic nanoparticles on an SiO_2_ carrier and exhibited a selectivity as high as that of the commonly used Lindlar catalyst (which contains such hazardous components as lead and barium), while the activity of the Fe-Pd-O/SiO_2_ catalyst was an order of magnitude higher. The hydrogenation of a triple bond over the proposed Pd-Fe catalyst opens the way to selective hydrogenation over nontoxic catalysts with a high yield and productivity. Taking into account a simple procedure of catalyst preparation, this direction provides a rationale for the large-scale implementation of these catalysts.

## 1. Introduction

Supported palladium catalysts are widely used for the selective hydrogenation of alkynes both in industry and in laboratory practice [1,2,3]. One of the most selective commercial catalysts of the liquid-phase hydrogenation to alkenes is a Lindlar catalyst that comprises a Pd catalyst partially poisoned with lead (5 wt.% palladium supported on calcium carbonate or barium sulfate and treated with various lead compounds [4]). The hydrogenation of alkynes, for the last decade, has been increasingly used in the synthesis of pharmaceuticals, vitamins, nutraceuticals, fragrances, and catalysts with no hazardous or toxic components, which are in strong demand. The economic reasons associated with the high palladium price and recent environmental regulations stimulate searching for nontoxic alternatives to the Lindlar catalyst as well. Several strategies have been applied [1], and most recent studies focus on the preparation of less toxic catalysts based on the supported modified Pd nanoparticles (NPs) with a high catalytic activity and selectivity at mild conditions in a three-phase liquid–solid–gas system [5,6,7,8,9,10,11,12,13,14,15,16,17]. Among the modifying additives in the catalyst developed, Ag, Au, Bi, C, Cu, Ga, Ni, Si, Sn, Ti, W, and Zn have been used [1,5,12,13,14,15,16,17]. Iron and its oxides could be the least toxic modifying additives, yet their effect was weak [15] or even negative [17] as compared with that of other elements. Different inorganic, hybrid, and organic materials have been studied as Pd catalyst supports, and SiO_2_ is one of the most appropriate carriers [10,11,12]. The catalytic activity of supported Pd nanoparticles in the selective liquid-phase hydrogenation depends both on the support nature and the conditions of catalyst preparation that affect the Pd particle size and electronic state [18]. The outstanding selectivity of the highly dispersed Pd mono and bimetallic nanoparticles is supposed to be due to suppressing the β-PdH phase formation [1,18]. The complete elimination of β-PdH formation was revealed in Pd-Fe/SiO_2_ systems of different compositions [19,20,21], yet they were not studied in alkyne hydrogenation. Also, the promoting effect of iron on palladium was observed in the hydrogenation of nitro compounds [22].

The selective hydrogenation of phenylacetylene (PhA) is considered as a convenient model reaction for the evaluation of catalysts for the selective hydrogenation of alkynes to alkenes under mild conditions [10,12,14,16]. On the other hand, phenylacetylene removal from styrene (St) feedstocks by selective liquid-phase hydrogenation is a process of great industrial importance [23,24].

One of the major problems in the hydrogenation of alkynes to alkenes over Pd catalysts is the drop of selectivity, even to zero, when approaching the high PhA conversion in the hydrogenation process. This makes it difficult to control the process, especially at high alkyne concentrations and in a large-scale production [8,12,13,16]. This phenomenon is especially pronounced for the Pd/SiO_2_ catalysts prepared on commercial SiO_2_ supports by simple, commercially scalable methods of ion exchange or impregnation followed by calcination [12]. In our previous publications [20,21], a new method of the synthesis of bimetallic Pd-Fe-O/SiO_2_ materials was presented. The catalysts consist of Pd^0^, Pd_1−x_Fe_x_, and FeO_x_ nanoparticles supported on silica. Some results on the liquid-phase hydrogenation of phenylacetylene to styrene with hydrogen at ambient conditions over the prepared materials have also been reported [25,26].

The goals of the present study are (i) to evaluate in detail the variation in the selectivity to styrene near the complete PhA hydrogenation over recently developed Pd-Fe-O/SiO_2_ catalysts prepared using a commercial support; (ii) to compare the catalytic properties of developed catalysts with a commercial Lindlar catalyst; (ii) to reveal the factors regulating the selectivity and the rate of St hydrogenation.

## 2. Materials and Methods

### 2.1. Preparation of the Materials

The supported nanoparticles were prepared by the deposition of the metal precursors on granulated (diameter 4–6 mm) commercial silica carriers with a high specific surface area S_BET_ = 220 m^2^g^−1^ (carrier HS in [20,21]). Commercial chemicals, (NH_4_)_3_Fe(C_2_O_4_)_3_·3H_2_O (98%, Acrus Organics) and [Pd(NH_3_)_4_]Cl_2_·H_2_O (41.42% Pd; Aurat, Russia), were used as metal precursors. The precursor deposition was performed by the method of incipient wetness impregnation, similar to the previously described procedure [21], at 60 °C (sample PdFe-1) or at a higher temperature of 80 °C (sample PdFe-2) instead of room temperature in order to avoid the formation of large crystallites on the surface of the support grain, which further resulted in the production of XRD detectable Pd^0^ nanoparticles [27,28]. Both metal precursors were introduced simultaneously in the same solution. Before impregnation, the carrier sample was dried in a rotor evaporator at 60 °C and 40 mbar. After impregnation for 16 h, the samples were dried in a rotor evaporator at 60 °C. The Pd and Fe loadings were the same in all samples (3 and 7.6 mass. % calculated for reduced samples). The prepared materials were denoted as PdFe-X-CA-RB, where “X” is the sample number, “CA” is the temperature of calcination, if any, and “RB” denotes the sample reduction (“R”—reduced with hydrogen at 400 °C or at “B” °C). The sample of the same composition described in our previous publications was studied in this work as well (PdFe-3). The commercial Lindlar catalyst (Acros Organics, Geel, Belgium) was used as a reference sample.

### 2.2. Characterization of the Materials

X-ray diffraction patterns were recorded using a DRON-2 diffractometer with Ni-filtered Cu Ka radiation, as described previously [20,21]. Diffuse reflectance infrared Fourier transform (DRIFT) spectra were recorded at room temperature (RT) using a NICOLET “Protege” 460 spectrometer (Madison, WI, USA) equipped with a diffuse reflectance attachment in the interval of 6000–400 cm^−^^1^ at a resolution of 4 cm^−^^1^ (500 scans). CaF_2_ powder was used as a reference. The fractioned sample (0.10–0.25 mm) was placed inside a special ampoule with a CaF_2_ window. Before the spectroscopic measurements, a sample was evacuated at 350 °C (or at 250 °C if the sample calcined at 250 °C was studied) for 120 min. CO was used as a probe molecule, and it was adsorbed at RT and an equilibrium pressure of 1000–1500 Pa. The spectra were recorded at RT. The calcined samples were evacuated again at a high temperature and treated at RT with hydrogen (6000 Pa) for 15 min. Then, hydrogen was outgassed at room temperature, and spectra of adsorbed CO were recorded. The assignment of bands was performed using published reviews [27,28].

X-ray photoelectron spectra were recorded using an ES-2403 spectrometer equipped with a PHOIBOS 100 MCD analyzer (Thermo Fisher Scientific, Waltham, MA, USA).

The microstructure and morphological characteristics of the samples were studied by scanning electron microscopy using a Hitachi SU8000 electron microscope (Tokyo, Japan). The samples were examined by X-ray microanalysis (SEM-EDX) using an Oxford Instruments X-max 80 energy dispersive X-ray spectrometer (Abingdon, UK) at an accelerating voltage of 20 kV and an operating distance of 15 mm.

The microstructure of the samples was studied by transmission electron microscopy (TEM) with a Hitachi HT7700 electron microscope (Tokyo, Japan). The images were taken in the light field mode at an accelerating voltage of 100 kV. The average size of the supported nanoparticles was calculated based on the analysis of 250–350 nanoparticles for each sample of the catalyst.

The characterization of the catalytic activity was performed in the model reaction of liquid-phase phenylacetylene (PhA) hydrogenation at room temperature and atmospheric hydrogen pressure, as described in our previous publications [25,26]. Initial specific reaction rates (r_0_) were calculated as moles of the substrate per mole of Pd per second at a conversion below 30%.

## 3. Results

### 3.1. Catalytic Activity of the Samples

**Figure 1:**
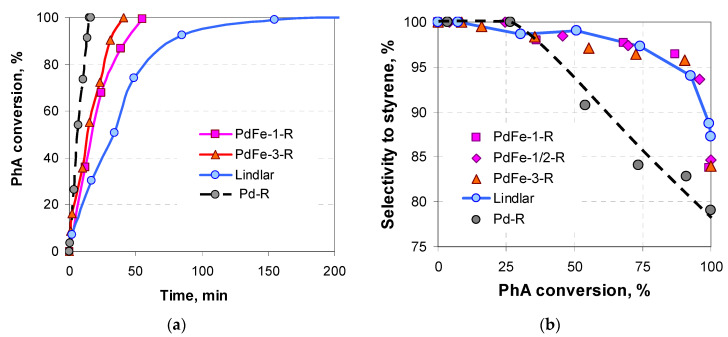
Time dependence of the phenylacetylene conversion (**a**) and variation in the selectivity to styrene vs. phenylacetylene conversion (**b**) over the samples prepared by the reduction of the dried supported precursors and over the Lindlar catalyst.

The prepared monometallic palladium catalyst Pd-R exhibits a high catalytic activity in the reaction of PhA hydrogenation at the chosen reaction conditions, the selectivity to styrene being quite high but significantly lower than that of the Lindlar catalyst (Figure 1). Moreover, the rate of St hydrogenation after reaching the complete PhA conversion is rather high (Table 1) with complete hydrogenation to EtB, as has already been pointed out [25]. At the same reaction conditions, there is no PhA adsorption or conversion over the monometallic iron oxide samples calcined at 250–400 °C or further reduced at 400 °C.

The bimetallic catalysts prepared by the reduction of the dried supported precursors are more selective but less active than the monometallic Pd catalyst (Figure 1, Table 1). Their selectivity dependences on the PhA conversion approach that of the Lindlar catalyst (Figure 1b), yet the prepared catalysts are considerably more active than the Lindlar catalyst. This is confirmed both by the higher initial rate of hydrogenation and the shorter time required for the complete PhA conversion (Table 1, Figure 1a). The good reproducibility of the results regarding the catalytic activity of the samples should be mentioned (points for PdFe-1-R and PdFe-1/2-R in Figure 1b), as well as the agreement between the values of the reaction rates of PhA uptake and hydrogen consumption (Table 1). During the reproduction of the samples, care should be taken regarding the conditions used for the support impregnation and further thermal treatment. Slight variation results in a strong difference in the PhA hydrogenation rate (Table 1, samples PdFe-1-R and PdFe-3-R).

The preparation procedure is supposed to be more essential for the catalytic properties than the structure of the silica support. It has been revealed that it is possible to prepare catalysts that exhibit high values of the selectivity to styrene at complete PhA conversion and a high catalytic activity in PhA hydrogenation to St combined with a low initial rate of further St hydrogenation by using another low-surface (LS) silica support (the surface area as low as 30 m^2^g^−1^; no micropores—LS in [20,21]) (Table 1, samples PdFe-4-R and PdFe-4-R-550Ar). When Pd-Fe/SiO_2_ samples are prepared by known procedures [19,22], the selectivity and activity are good enough (Table 1, sample PdFe-5-C450-R440), yet the rate of St conversion is high, and significant Pd and Fe leaching is observed.

**Figure 2:**
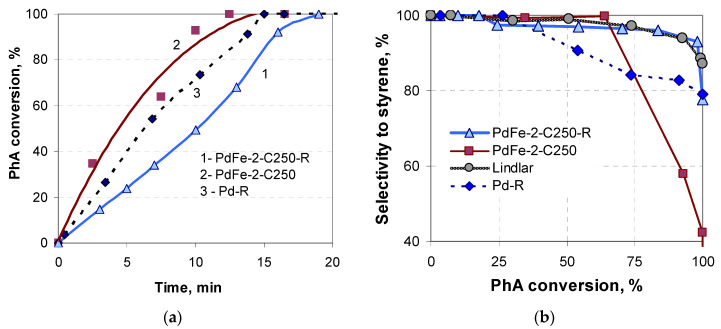
Time dependence of the phenylacetylene conversion (**a**) and variation in the selectivity to styrene vs. phenylacetylene conversion (**b**) over the sample reduced after calcination and the reference catalysts.

The bimetallic samples synthesized by the thermal decomposition of dried supported precursors in air at 250 °C (the minimal temperature of their decomposition found in our previous studies [20,21]) exhibit a higher activity as compared with the monometallic Pd catalyst (Figure 2a), yet their selectivity is low (Figure 2b). The reduction of these samples in a hydrogen flow at 400 °C results in a slight decrease in the activity, yet the selectivity to styrene increases to the values typical for the Lindlar catalyst (Figure 2). The reduced catalyst PdFe-2-C250-R is more selective than the monometallic Pd catalyst (Figure 2b, Table 1) and considerably more active than the Lindlar catalyst. The values of the St mass produced over 1 g of the catalyst per hour is an order of magnitude larger for the prepared reduced catalysts as compared with the Lindlar catalyst (Table 1) and may be increased by 60–100% while varying the reduction temperature, PhA:Pd ratio, and PhA concentration. For example, at PhA:Pd = 950 and C = 0.167 M, the sample PdFe-2-C250-R productivity is 34 g_St_·g_cat_^−1^·h^−1^ at a selectivity of 86%.

**Figure 3:**
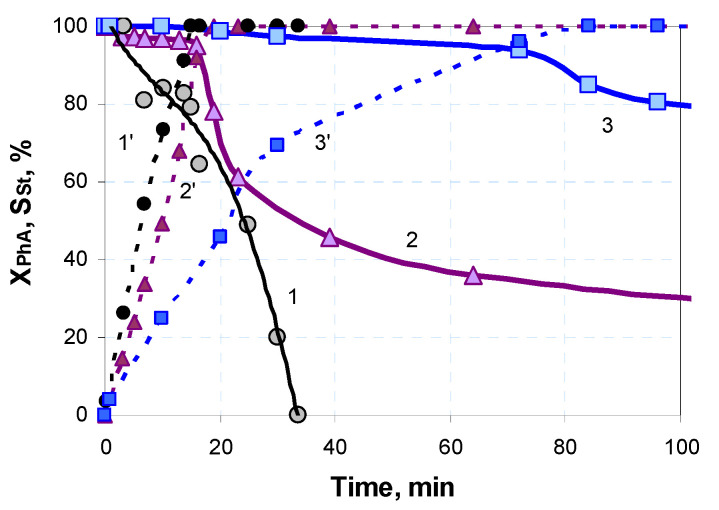
Time dependence of the selectivity to styrene and phenylacetylene conversion (dashed lines) over the catalysts Pd-R (1,1′), PdFe-2-C250-R (2,2′), and PdFe-1-R (3,3′).

The advantage of bimetallic Pd-Fe catalysts prepared by the reduction of the dried supported precursors is the lower rate of St hydrogenation at complete PhA hydrogenation (Table 1). The slow St hydrogenation extends the period with a high selectivity (Figure 3) and could make it possible to achieve and maintain a high selectivity when the process is to be scaled up.

**Figure 4:**
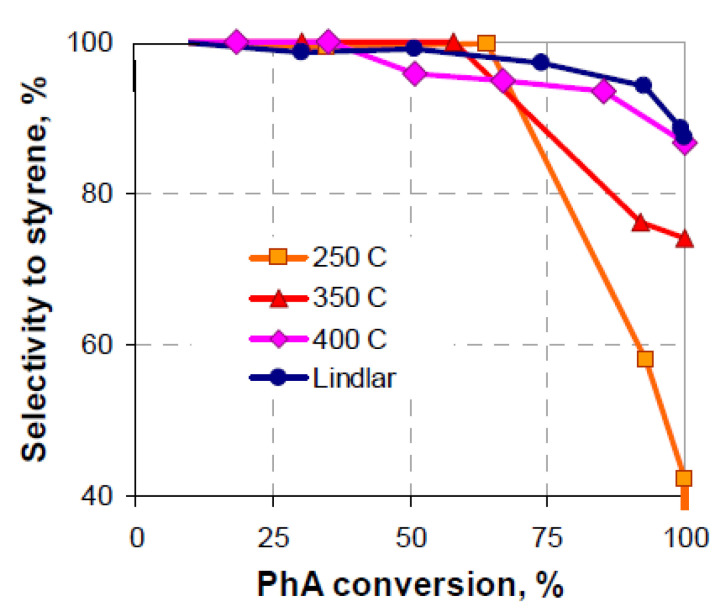
Variation in the selectivity to styrene vs. phenylacetylene conversion over the sample PdFe-2 calcined at different temperatures and over the Lindlar catalyst.

In our recent studies [25,26], we revealed that the Pd-Fe samples calcined in an air flow catalyze both PhA and St hydrogenation, even with no preliminary reduction of the sample. The catalytic activity is comparable to or exceeds the activity of the reduced monometallic Pd catalyst with the same Pd loading. The catalytic properties depend on the temperature of calcination. In the present study, it is shown that, among the bimetallic catalysts with a Pd loading of 3%, the most active is the sample calcined at 350 °C, yet its selectivity is not high enough at the conversion values approaching the complete PhA conversion in hydrogenation (Figure 4). The selectivity to St increases with the calcination temperature being up to 400 °C and becomes comparable with the selectivity of the Lindlar catalyst (Figure 4). However, in contradiction to the preliminary reduced samples, the calcined samples exhibit a high rate of styrene hydrogenation to ethylbenzene, increasing with the calcination temperature from 0.46 s^−1^ to 1.0 s^−1^. On the other hand, the value of the St mass produced over 1 g of the catalyst per hour is 31, i.e., 1.5–2 times larger than that for other selective Pd-Fe catalysts (Table 1) and 15 times larger than that for the Lindlar catalyst.

**Figure 5:**
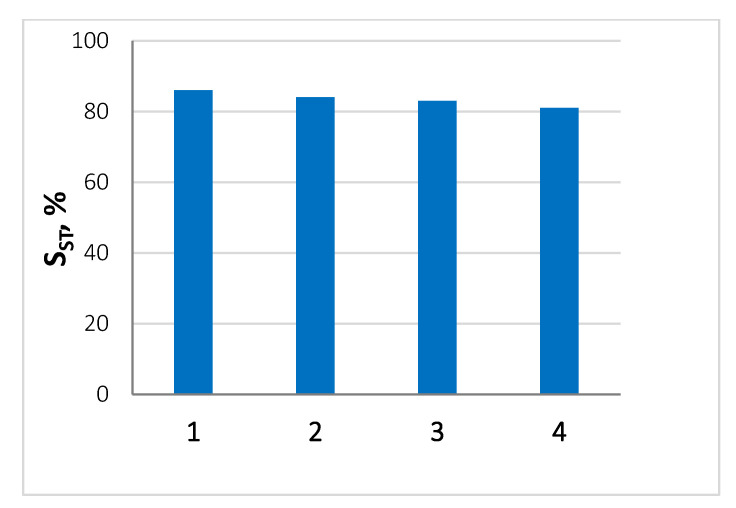
The stability of the PdFe-2-C400 sample, expressed in terms of the selectivity of styrene formation at full conversion.

As can be seen from Figure 5, the sample PdFe-2-C400 has a high stability and does not deactivate during four reaction cycles without first washing it from the reaction medium. This confirms the fact that there is no leaching of the active phase and deactivation of the catalyst by reaction products.

Considering a significant difference in the catalytic behavior of the Pd-Fe/SiO_2_ samples with the same composition and starting steps of preparation, it seems important to gain an understanding of the Fe influence on the Pd active surface sites and the effect of preparation variables on it.

### 3.2. Characterization of the Samples

**Figure 6:**
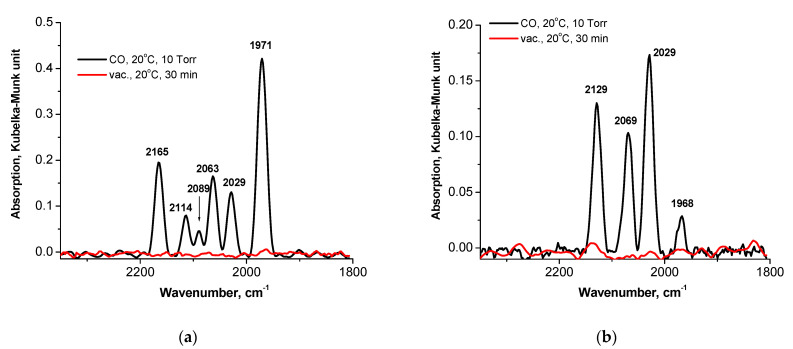
DRIFT spectra recorded after CO adsorption on the PdFe-3-R (**a**) and PdFe-2-C250-R (**b**) samples (preliminarily reduced in hydrogen at 400 °C).

**Figure 7:**
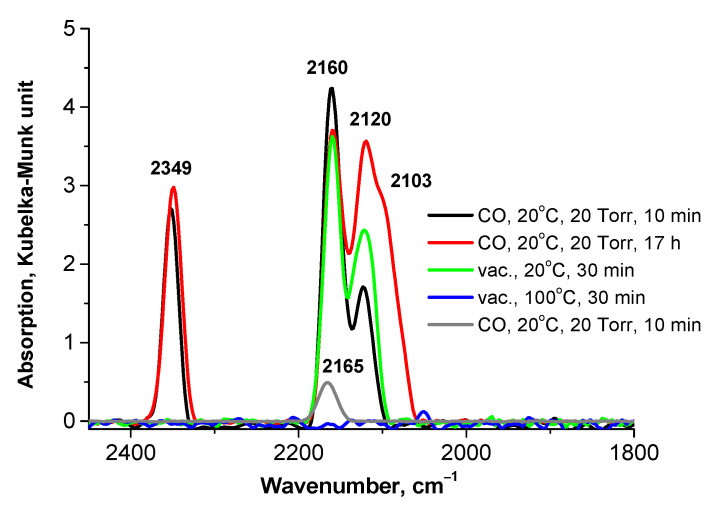
DRIFT spectra recorded after CO adsorption on the calcined PdFe-2-C350 sample.

**Figure 8:**
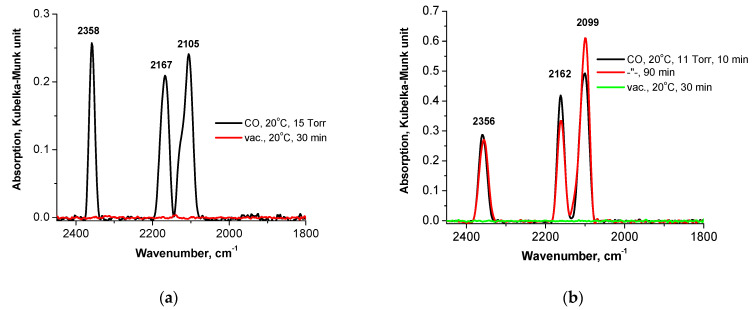

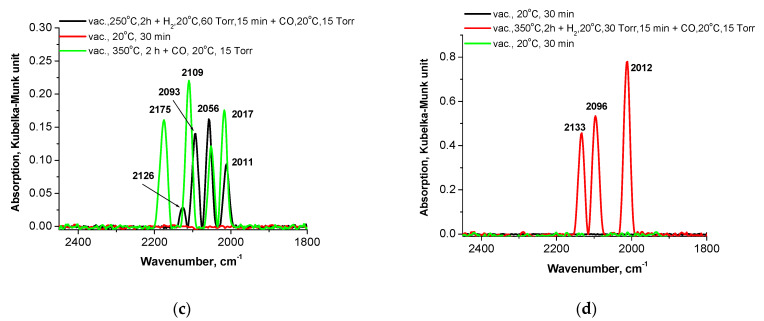
DRIFT spectra recorded after CO adsorption on the calcined PdFe-2-C250 (**a**,**c**) and PdFe-2-C400 (**b**,**d**) samples before (**a**,**b**) and after in situ treatment with H_2_ at room temperature (**c**,**d**).

The reduced monometallic Pd/SiO_2_ sample contains Pd^0^ crystallites of an average particle size of 25 nm. The supported nanoparticles in the bimetallic samples prepared in the present work are X-ray amorphous, except for the sample PdFe-2-C250-R, reduced after calcination. The Pd-Fe samples prepared previously are characterized by a number of physico-chemical methods in our publications [20,21,25,26], and the results are summarized in Table 2. The feature of these Pd-Fe samples is the presence of nano crystallites of both Pd^0^ and Pd_1−x_Fe_x_ or Fe^0^ and Pd_1−x_Fe_x_ phases. In the present work, to study the state of metals on the surface, DRIFT spectroscopy of chemisorbed CO is applied, and the obtained spectra are depicted in Figure 6, Figure 7 and Figure 8.

#### 3.2.1. Preliminarily Reduced Samples

There are several bands in the spectral ranges of surface Pd carbonyls in DRIFT spectra recorded after CO adsorption on the samples reduced preliminarily in a hydrogen flow at 400 °C, yet the spectra differ depending on whether a dried or calcined sample is reduced (Figure 6). The bands of linear complexes are observed in both spectra at 2114–2129 cm^−1^ (Pd^+^—CO complexes) and at 2063–2089 cm^−1^ (Pd^0^—CO complexes), exhibiting the high dispersion of Pd and its strong interaction with Fe species. The appearance of the Pd^+^—CO band in the samples reduced in hydrogen at such a high temperature may be due to the formation of Pd^n+^-Fe^2+^ species that further crystallize into alloy Pd_1−x_Fe_x_ after annealing in Ar at 550 °C [20]. The bands of Pd^0^—CO complexes at 2084 cm^−1^ were observed by another research group for the silica-supported Pd-Fe samples after a reduction in hydrogen at 300 °C, the red shift from 2084 to 2064 being revealed with an increase in the Fe/Pd ratio [22]. The bands are assigned to CO adsorbed on on-top sites exposed at (111) microfacets.

The intensive band at 1971 cm^−1^ of bridging carbonyls Pd^+^—CO—Pd^+^ assigned to CO adsorbed on the (100) sites [22] indicates the presence of a considerable number of large-enough Pd nanoparticles in the sample PdFe-3-R prepared by the reduction of dried supported precursors, which agrees with the results of EXAFS and XRD studies (Table 2). The intensity of this band and its ratio to the intensity of the bands of linear complexes are an order of magnitude lower in the spectra of the sample PdFe-2-C250-R, supposing the higher Pd dispersion in the sample reduced after calcination, which looks contradictory to the results presented in a recent paper [21]. The only reason for this difference may be the difference in the preparation procedure, namely, the higher temperature of the support impregnation and drying used for the preparation of PdFe-2. The conclusion follows from this observation: the formation of large Pd^0^ nanoparticles occurs via the fast sintering of metallic species generated under the reductive decomposition of supported precursors in hydrogen. Decomposition in hydrogen at low temperatures of 250 °C supposes a smaller nuclei size than the reduction of oxide to metal and, therefore, higher rates of sintering and particle growth. The absence of the band at 1971 cm^−1^ in the spectra of calcined samples (Figure 7 and Figure 8) is commonly considered to be proof of the high Pd^0^ dispersion, or it can be an indication that iron atoms or ions are preferentially located on the (100) sites of Pd^0^ nanoparticles [22]. It is known that the higher alkene selectivity of the Pd nanocubes is attributed to the large adsorption energy of the carbon–carbon triple bond on the {100} facets [29].

The presence of Fe^2+^ in the sample PdFe-3-R is confirmed by the presence of the band at 2165 cm^−1^, although it is absent in the spectra of the PdFe-2-C250-R sample. The latter fact may be explained both by the higher extent of Fe^n+^ reduction enhanced by the presence of Pd^0^ nanoparticles formed upon calcination and by the enrichment of the surface of bimetallic nanoparticles and Fe_3_O_4_ crystallites with Pd^0^ atoms (generation of core-shell structures). This band is not observed in the spectra of Pd-Fe/SiO_2_ bimetallic catalysts prepared by other procedures [22].

#### 3.2.2. Preliminarily Calcined Samples

The adsorption of CO on the oxidized Pd-containing samples in a CO atmosphere is almost always accompanied by a reduction of Pd^n+^ to Pd^0^, which is indicated by the appearance of bands at wavenumbers below 2100 cm^−1^ in the IR spectra of adsorbed CO. This phenomenon is observed for the calcined samples (Figure 7 and Figure 8a,b), especially at the prolonged treatment of the PdFe-2-C350 sample with CO (Figure 7). The appearance of the band of adsorbed CO_2_ at 2349 cm^−1^ [28] is an indication of the oxidation of CO adsorbed on Fe^3+^ ions with lattice O^2−^ ions. The band at 2160 cm^−1^ may be attributed to the linear form of CO adsorption on Fe^2+^, but the intensity of this band at 2165 cm^−1^ in the sample containing no Pd is considerably lower (Figure 7, grey line). It should be mentioned that its intensity decreases slightly in the spectra of bimetallic samples upon exposition to CO (Figure 7 and Figure 8b). Therefore, it seems preferable to attribute the bands at 2160 cm^−1^ and 2120 cm^−1^ to the symmetric and antisymmetric stretching vibrations of Pd^2+^(CO)_2_ complexes, respectively. The disappearance of the band at 2162–2167 cm^−1^ after the sample treatment with hydrogen at room temperature (Figure 8c,d) confirms such an assignment of the bands. In the case of the samples calcined at 250 and 400 °C that strongly differ in the selectivity to styrene (Figure 4), DRIFT spectroscopic studies with adsorbed CO reveal at least two types of Pd^2+^ surface sites that can be easily reduced with hydrogen even at room temperature, resulting in the formation of Pd^+^ (the bands at 2125 cm^−1^ and 2133 cm^−1^) and two states of Pd^0^ (the bands at 2093–2096 cm^−1^ and 2012 cm^−1^). The bands at 2063–2089 cm^−1^ can be attributed to Pd^0^—CO complexes exhibiting the high dispersion of Pd and its strong interaction with Fe species. The band at 2012 cm^−1^ was not observed previously for any Pd-Fe/SiO_2_ systems [22,27]. This band may be assigned to CO adsorbed on the (100) Pd sites affected by interaction with the Fe_2_O_3_ surface. As follows from X-ray photoelectron spectroscopy and theoretical calculation results [29,30,31], the interaction between Pd and the Fe_2_O_3_ surface occurs through the exchange of electrons with the surface Fe and O atoms. This bonding between Pd and surface oxide elements causes Pd to partially donate electrons to the oxide surface. This may be the reason for the blue shift of the band of bridging carbonyls Pd^+^—CO—Pd^+^ from 1971 cm^−1^ to 2011–2017 cm^−1^. One more state of Pd^0^ (the band at 2056 cm^−1^) is revealed for the sample calcined at 250 °C. Thus, formed Pd^0^ sites should be present on the surface before the introduction of PhA, and therefore, they participate in the reaction process. The Pd^0^ sites (the band at 2011–2017 cm^−1^) formed in the calcined samples during reduction with H_2_ at RT seem to be responsible for St hydrogenation. The most selective sample, PdFe-2-C400, contains neither Fe^2+^ nor Pd^0^ sites, which are specific for the band at 2056 cm^−1^ after treatment with H_2_. This fact makes us suppose their responsibility for the direct PhA hydrogenation to ethylbenzene and the low selectivity to styrene. After reduction in hydrogen at RT, the Pd^0^ sites seem to be partly blocked with H_2_O molecules, whereas Fe^2+^ are completely blocked. The increase in the intensity of the Pd^0^ bands and the appearance of the Fe^2+^ band at 2176 cm^−1^ after evacuation at 350 °C (Figure 8c) prove this assumption. Such a blocking effect can modify the catalytic properties as well.

#### 3.2.3. XPS Investigation 

**Figure 9:**
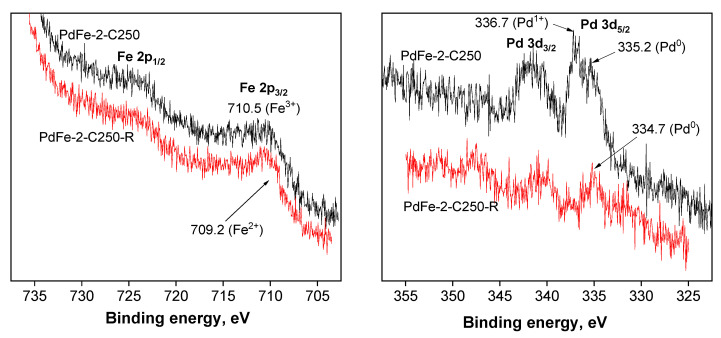
XPS data for PdFe-2 catalysts.

The study of the catalysts PdFe-2-C250 and PdFe-2-C250-R by the XPS method showed that, depending on the conditions of the heat treatment, both the electronic state of the supported metal nanoparticles and the distribution of metals in the surface layer of catalysts changed. Figure 9 shows the photoelectron spectra in the Fe 2p region. The energy position of Fe 2p_3/2_ at 710.5 eV and the shape of the Fe 2p doublet lines of photoelectrons in the spectra of both samples differ. In the spectrum of PdFe-2-C250-R, a shoulder is observed from the side of lower binding energies at 709.2 eV, which indicates the presence of mainly the FeO oxide in the reduced sample, while the oxidation degree of Fe^3+^ is characteristic of the calcined sample of PdFe-2-C250 [32]. The increase in the Fe/Si atomic ratio during the reduction in the catalyst by almost 1.5 times may be due to the segregation of iron in the surface layers of this sample (Table 3). An analysis of the X–ray spectra in the Pd 3d region of photoelectrons (Figure 9) at 336.7 and 335.2 eV allows us to conclude that palladium in the surface layers of the sample PdFe-2-C250 exists in two oxidation states, Pd^1+^ and Pd^0^, respectively [33,34]. According to the XPS data for the sample PdFe-2-C250-R, all of the palladium is present in a metallic state [34]. The energy position of Pd 3d_5/2_ electrons and the atomic ratio Pd/Si are presented in Table 3.

#### 3.2.4. SEM and TEM

**Figure 10:**
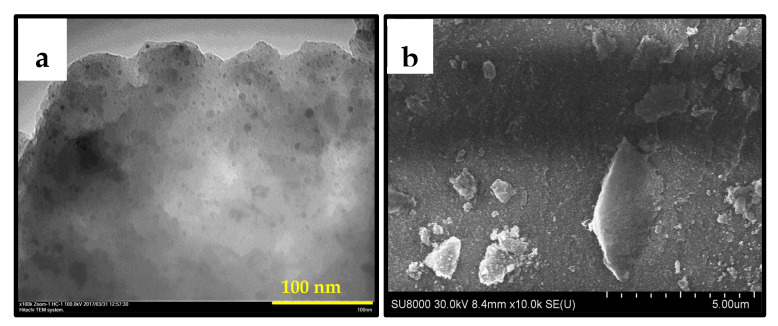
Micrographs of TEM (**a**) and SEM (**b**) of the PdFe-2-C250 catalyst.

The morphological characteristics of bimetallic PdFe-2 catalysts that have undergone various heat treatments have been studied by TEM and SEM methods. The TEM micrograph (Figure 10a) of the sample PdFe-2-C250 shows spherical particles of an average diameter of 5 nm, evenly distributed over the surface of the carrier. On the SEM micrograph (Figure 10b) of this sample, the crystalline phase of iron oxides can be noted.

**Figure 11:**
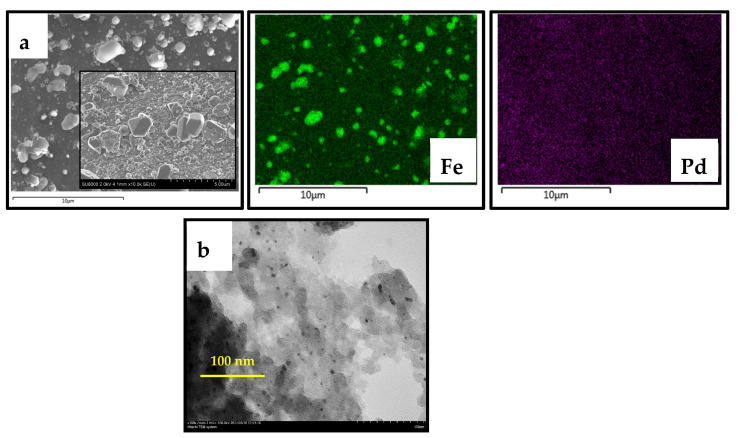
Micrographs of SEM-EDX (**a**) and TEM (**b**) of the PdFe-2-C250-R catalyst.

Micrographs were also obtained for the PdFe-2-C250-R sample with its subsequent reduction in an H_2_ flow at 400 °C. The SEM image (Figure 11a) clearly shows crystals of an ordered structure. SEM-EDX data show that these crystal structures consist mainly of iron atoms. Palladium is evenly distributed over the entire surface of the carrier in the form of much smaller particles, which also include iron. It should be noted that the particles in the reduced sample PdFe-2-C250-R are evenly distributed over the entire surface of the carrier, and the average size of the nanoparticles is 5 nm (Figure 11b).

## 4. Conclusions

Thus, based on our investigation of the electronic state of Pd and Fe metals in calcined bimetallic PdFe-2 catalysts by CO chemisorption (DRIFTS-CO), the existence of a strong contact interaction due to the electronic effect between Pd (Pd^+^, Pd^0^) and Fe (Fe^2+^, Fe^3+^) has been established. By the TEM method, it was found that the addition of iron to palladium nanoparticles in the PdFe-2 catalysts leads to a significant decrease in the aggregation of crystallites and an increase in the dispersion of the active metal relative to the monometallic palladium Pd-R catalyst [25]. In conclusion, the high dispersed phase, the strong contact interaction of the metals Pd-Fe, and the absence of palladium hydride formation in bimetallic catalysts lead to the competitive adsorption of the C≡C bond on the surface of bimetallic PdFe-2 catalysts, which is the reason for the high catalytic activity in the selective hydrogenation of phenylacetylene to styrene with a high selectivity of 84–90% at the complete conversion of phenylacetylene. It has been shown that the formation of nanoparticles of the solid solution Pd_1−x_Fe_x_ is essential for the selective hydrogenation in order to considerably decrease the rate of styrene hydrogenation to ethylbenzene.

The bimetallic Pd-Fe/SiO_2_ catalysts have wide potential practical implications. At least three relatively simple preparation procedures can be used for their preparation: (i) the direct reduction of silica-supported metal precursors, (NH_4_)_3_[Fe(C_2_O_4_)_3_] and [Pd(NH_3_)_4_]Cl_2_, with a hydrogen flow at 400 °C; (ii) the thermal decomposition of the silica-supported metal precursors in air at 250 °C followed by a reduction in the hydrogen flow at 400–430 °C; (iii) the thermal decomposition of silica-supported metal precursors in air at 400 °C. Considering the strong sensitivity of the selectivity and reaction rate to the preparation variables, care should be taken when scaling up the catalyst production. The choice of the preparation procedure will depend on environmental regulations, technical and economical possibilities, as well as the principal direction of implementation.

Summarizing the results obtained, the prepared bimetallic Pd-Fe/SiO_2_ catalysts can be proposed as a new non-toxic alternative to the Lindlar catalyst for the selective hydrogenation of triple C≡C bonds in the liquid phase at room temperature and atmospheric hydrogen pressure. These novel catalytic materials are as selective in the hydrogenation of the triple C≡C bond as the commercial Lindlar catalyst, and their productivity values are up to an order of magnitude higher.

## Figures and Tables

**Table 1:** The catalytic activity of the samples reduced in a hydrogen flow at 400 °C.

Sample	r_St_ ^a^, s^−1^	Ƞ_St_ ^b^_,_ g_St_·g_cat_^−1^·h^−1^
PdFe-1-R	0.02	5.7
PdFe-1/2-R ^c^	0.05	4.5
PdFe-2-C250-R	0.20	17
PdFe-2-C250-R430 ^d^	0.0025	34
PdFe-2-C250	0.46	15
PdFe-2-C350	0.62	48
PdFe-2-C400	1.0	31
Pd-R	0.51	23
Pd-R ^e^	0.53	23
PdFe-3-R	0.02	14
Lindlar catalyst	<0.000	2.0

Reaction conditions: 0.13 M PhA, molar ratio PhA: Pd = 300 ± 20; ^a^ selectivity to styrene at complete PhA conversion; ^b^ r_o_—the initial rates of PhA uptake and H_2_ consumption; ^c^ St mass produced over 1 g of catalyst per hour; ^d^ r_St_—the rate of St hydrogenation after the complete PhA conversion; ^e^—repeated test.

**Table 2:** The surface sites in supported nanoparticles revealed with DRIFT spectroscopy and the average particle size and phase based on the XRD method [21,22].

Sample	Possible Surface Sites of Pd and Fe	Phase and Average Particle Size, nm
PdFe-2-C250-R	Pd^0^ (top > facet cites), Pd^+^	XRD: Pd^0^-10 nm, Fe_3_O_4_-17 nm TEM: isolated nanoparticles (<5 nm)
PdFe-2-C250	Pd^2+^ reducible to Pd^+^ with CO or to Pd^0^ with H_2_ at RT; Fe^2+^, Fe^3+^	XRD: Amorphous TEM: isolated nanoparticles (<5 nm)
PdFe-2-C350	Pd^2+^ reducible to Pd^+^ with CO at RT; Fe^2+^, Fe^3+^	XRD: Amorphous
PdFe-2-C400	Pd^2+^ reducible to Pd^0^ with CO or both to Pd^+^ and Pd^0^ with H_2_ at RT; Fe^2+^, Fe^3+^	XRD: Amorphous
PdFe-3-R	Pd^0^ (I: facet > top > X1 cites), Pd^+^, Fe^2+^	XRD: Pd^0^ (15 nm)
Pd-R	Pd^0^	XRD: Pd^0^ (25 nm)

**Table 3:** Binding energies and atomic ratios according to XPS data.

Sample	Binding Energy, eV		Surface Atomic Ratio			Atomic Ratio
	Fe 2p_3/2_	Pd 3d_5/2_	Fe/Si	Pd/Si	Fe/Pd	Fe/Pd
PdFe-2-C250	710.5	336.7 335.2	0.0094	0.0091	1.03	2.67
PdFe-2-C250-R	710.3 709.2	334.7	0.0135	0.0013	1.03	2.67

## Data Availability

Data are available from the authors upon request.
